# Caution is required in the implementation of 90-day mortality indicators for radiotherapy in a curative setting: A retrospective population-based analysis of over 16,000 episodes

**DOI:** 10.1016/j.radonc.2017.07.031

**Published:** 2017-10

**Authors:** K. Spencer, R. Ellis, R. Birch, E. Dugdale, R. Turner, D. Sebag-Montefiore, G. Hall, A. Crellin, E. Morris

**Affiliations:** a Leeds Cancer Centre, Leeds Teaching Hospitals NHS Trust, St James’s University Teaching Hospital, Leeds, UK; b Cancer Epidemiology Group, Leeds Institute of Cancer and Pathology, University of Leeds, Level 6, Leeds, UK; c Radiotherapy Research Group, Leeds Institute of Cancer and Pathology, University of Leeds, Leeds Teaching Hospitals NHS Trust, St James’s University Teaching Hospital, Leeds, UK

**Keywords:** Radiotherapy, Clinical indicator, Radical, Outcome, Mortality

## Abstract

**Background:**

90-day mortality (90 DM) has been proposed as a clinical indicator in radiotherapy delivered in a curative setting. No large scale assessment has been made. Its value in allowing robust comparisons between centres and facilitating service improvement is unknown.

**Methods:**

All radiotherapy treatments delivered in a curative setting over seven years were extracted from the local electronic health record and linked to cancer registry data. 90 DM rates were assessed and factors associated with this outcome were investigated using logistic regression. Cause of death was identified retrospectively further characterising the cause of 90 DM.

**Results:**

Overall 90 DM was 1.25%. Levels varied widely with diagnosis (0.20–5.45%). Age (OR 1.066, 1.043–1.073), year of treatment (OR 0.900, 0.841–0.969) and diagnosis were significantly associated with 90 DM on multi-variable logistic regression. Cause of death varied with diagnosis; 50.0% post-operative in rectal cancer, 40.4% treatment-related in head and neck cancer, 59.4% disease progression in lung cancer.

**Conclusion:**

Despite the drive to report centre level comparative outcomes, this study demonstrates that 90 DM cannot be adopted routinely as a clinical indicator due to significant population heterogeneity and low event rates. Further national investigation is needed to develop a meaningful robust indicator to deliver appropriate comparisons and drive improvements in care.

90 day mortality has been suggested in the English NHS as a clinical indicator following radiotherapy delivered in a curative setting (RDCS) [1]. It is proposed that this will deliver comparative assessments of quality of care across providers. Such assessments, aiming to inform patient choice and support service improvement [2], are now routinely used in surgery [3], [4], [5], [6] and are increasingly seen across a range of other healthcare interventions, including chemotherapy [7], [8], [9]. It has been shown, however, that in settings where rates of early mortality are low, and where procedures are infrequent, indicators may not be adequately powered to identify outlying practice [10], [11]. This may result in failure to identify poorly performing centres, complacency amongst those wrongly identified as performing in line with expectations and significant reputational damage and patient anxiety in centres falsely identified as underperforming. In addition, the ambition to transform services into learning organisations [12] depends upon the availability of indicators to quantify and understand variations in care and outcomes.

In this context it is vital to ensure the indicators used are appropriate. A number of requirements must be met to ensure this: data must be robust; the population relatively homogeneous; the indicator must reflect quality and be adequately powered to identify outlying practice. Failure to meet these objectives may render them at best unhelpful and at worst counter-productive.

Approximately 65,000 radiotherapy treatments are delivered in England each year in a curative setting [13]. Treatment courses range from short pre-operative, definitive longer course radiotherapy or chemo-radiotherapy, through to post-operative adjuvant radiotherapy. The toxicities of these complex pathways and the populations treated within them vary widely. Where significant toxicity is experienced quality supportive care is key to ensuring good outcomes and the avoidance of harm. Unfortunately, for some patients it is disease progression, during or very shortly after treatment that results in death. Identifying the primary cause of mortality is, however, complex and it is unclear if a single indicator has value across all treatment approaches.

To date only small scale assessments of 90 DM following RDCS have been carried out with overall rates of around 2.3% reported [14]. With this evidence it is unclear if the 90 DM indicator can meet the required standards to ensure valid, clinically meaningful outcomes in this setting.

This study aimed to investigate 90 DM in a large 7-year regional cohort in England. It assessed the factors associated with 90 DM, considered the value of this indicator in guiding service improvements and investigated its potential to provide robust comparisons between centres.

## Materials and methods

All radiotherapy episodes delivered in Leeds Cancer Centre (LCC), between January 2004 and December 2010, were identified using the electronic patient record. Patient demographics (date of birth and sex) and treatment information (date of treatment, planned fractionation, dose, treatment intent and site treated) were extracted from this resource. These data were linked to the cancer registrations held by the National Cancer Registration and Analysis Service (Northern and Yorkshire), ensuring robust diagnostic, socioeconomic status (SES) and date of death information were available for all linked records. SES was derived on the basis of rank quintile of the Index of Multiple Deprivation (IMD), (ONS 2010 version) [15], for the Lower Super Output Area (population defined geographical region of approximately 1500 people [16]) of residence at diagnosis.

Diagnosis was defined using International Classification of Diseases (ICD-10) codes [17]. Clinically recognised diagnostic groups were formed by combining diagnoses; Brain tumours included all central nervous system tumours, Head and Neck (H + N) cancer encompassed all cancers arising between the hypopharynx (inferiorly) and nasopharynx (superiorly) and salivary gland tumours, excluding sarcomas. See supplementary Table 1s for ICD10 groupings. A significant number of patients had multiple malignant diagnoses and were identified as such. Small diagnostic groups were combined to form the “Other” category, this included, but was not limited to, thyroid cancer, and male and female genital tract tumours not otherwise classified.

**Fig. 1:**
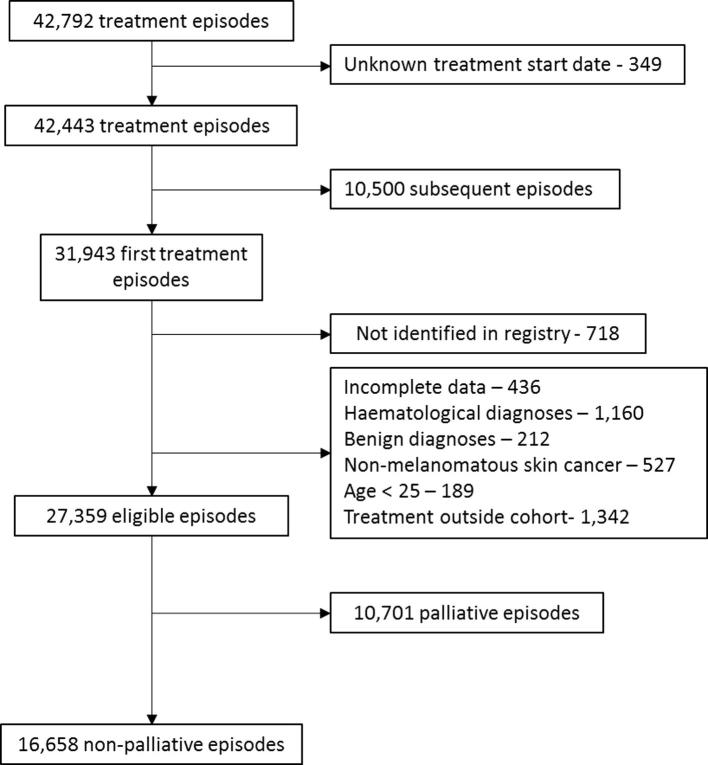
Consort diagram illustrating exclusions to reach final study population.

Intent of treatment was defined using a combination of treatment dose, fractionation, intent specified by clinicians and departmental protocols. RDCS included all neo-adjuvant, adjuvant and primary radiotherapy/chemo-radiotherapy. Throughout the study period, treatment was delivered within well-defined clinical protocols with limited change over time. To ensure that patients only entered the cohort once and that fragmented courses (e.g. where 2 phases were recorded separately) were not considered twice, only the first episode was considered. Exclusions were made to limit this investigation to adult RDCS treatments, for solid organ tumours and to ensure data quality (Fig. 1). Patients under the age of 25 are treated within the paediatric and young adolescent practice and were therefore excluded.

LCC is a university affiliated centre serving a population of 2.8 million (the second largest UK radiotherapy centre). Consultant clinical oncologist numbers increased from 18 to 30 during the study period. All are site specialised to a maximum of three primary diagnostic groups. LCC is resourced through a national NHS tariff system (reflecting treatment planning complexity and separately the number of fractions and complexity of delivery).

### 90-day mortality

The proportion of people dying within 90-days of the start of treatment was assessed. The start of treatment was used as the reference date providing a consistent time point across all fractionation patterns delivered, ensuring capture of deaths occurring on treatment and aligning with the methodology used in other interventions [18]. The dependent variable, death within 90-days, was considered as a binary outcome. Factors potentially impacting upon 90 DM were considered using logistic regression. Explanatory variables included, age at the start of radiotherapy (a continuous variable), sex, socioeconomic status, primary diagnosis and year of treatment. Colorectal cancer was used as the baseline diagnostic group within the logistic regression model, representing the largest disease group including both male and female patients across a wide range of age and SES. Patients in whom the SES was not known (506 individuals) were omitted from regression analysis.

### Cause of death (COD)

For all patients dying within 90 days of the start of radiotherapy COD was determined (malignancy, treatment, co-morbidity or post-operative) using death certificate data in combination with retrospective clinical record review. This assessment was made in order to determine what is measured by 90 DM and, hence, the quality of the indicator. Determining the underlying COD can be challenging. COD was assessed by two investigating clinicians independently to provide as accurate an assessment as possible.

Further investigation of the H + N and lung cancer populations was carried out. These two groups were considered due to their size and the moderate 90 DM rates seen, allowing more in depth analysis incorporating the impact of anatomical subsite (oropharyngeal versus other H + N sites), morphology (non-small cell versus small cell lung cancer) and year of treatment. The introduction of the cancer waiting times directive within the NHS in the early part of this cohort [19] and increased capacity within the service in 2008 resulted in a marked reduction in waiting times. Consistent information on waiting time was only available for the first four years. Time from decision to treat to first treatment (TTFT) was determined for this cohort and variation between years assessed using ANOVA.

Statistical analyses were carried out using STATA IC 14. The study was approved by the local audit department.

## Results

The final study population consisted of 16,675 radiotherapy treatments. Women were the majority of the cohort (10,541 (63.2%)), reflecting the large number of patients (6597 (39.6%)) treated for breast cancer. Prostate cancer (1993 (12%)), colorectal cancer (1197 (7.2%)), H + N cancer (1165 (7.0%)) and lung cancer (871 (5.2%)) were the next most frequently treated diagnoses. The distribution of age and SES were in line with expectations. The number of treatments delivered each year rose from 2001 to 2699 between 2004 and 2010 (see Table 1).

**Table 1:** Characteristics and 90 day mortality of the treated population.

	Total treated	%	Deaths within 90 days	90 DM
*Sex*				
Female	10,541	63.28	84	0.80
Male	6117	36.72	124	2.03
*Age*				
≤50	2934	17.61	13	0.44
51–60	3995	23.98	33	0.83
61–70	5353	32.13	50	0.93
71–80	3451	20.72	62	1.80
>80	925	5.55	50	5.41
*IMD*				
Most deprived	3257	19.55	55	1.69
4	2892	17.36	47	1.63
3	2812	16.88	31	1.10
2	3808	22.86	40	1.05
Most affluent	3383	20.31	30	0.89
Unknown	506	3.04	5	0.99
*Diagnosis*				
Colorectal	1197	7.19	31	2.59
Anal	166	1.00	3	1.81
Bladder	353	2.12	15	4.25
Brain	303	1.82	6	1.98
Breast	6596	39.6	22	0.33
Cervical	300	1.8	3	1.00
Lung	871	5.23	31	3.56
H + N	1165	6.99	43	3.69
Multiple	2325	13.96	28	1.20
Oesophageal	202	1.21	11	5.45
Prostate	1993	11.96	4	0.20
Sarcoma	145	0.87	1	0.69
Uterine	327	1.96	2	0.61
Other	715	4.29	8	1.12
*Year*				
2004	1996	11.98	27	1.35
2005	2242	13.46	45	2.01
2006	2555	15.34	33	1.29
2007	2320	13.93	34	1.47
2008	2422	14.54	19	0.78
2009	2425	14.56	21	0.87
2010	2698	16.2	29	1.07
Total	16,658		208	1.25

Overall, 90 DM was 1.25%, but varied widely with diagnosis ranging from 0.2% in prostate cancer to 5.45% in oesophageal cancer. Lung (3.89%) and H + N cancers (3.86%) had moderate levels of 90 DM (see Table 1).

Factors significantly associated with increased 90 DM on univariable logistic regression included increasing age, earlier year of treatment and individual diagnostic groups (see Table 2). Age and year retained their significance on multivariable analysis. Breast (OR 0.248, *p* < 0.001) and prostate (OR 0.076, *p* < 0.001) cancer treatments were associated with significantly lower 90 DM than colorectal cancer whilst head and neck cancer (OR 1.837, *p* = 0.014) treatment was associated with significantly higher 90 DM.

**Table 2:** Univariable and multivariable logistic regression of 90 day mortality and case-mix factors.

Co-variable	Univariable logistic regression			Multivariable logistic regression		
	OR	*p*	95% CI	OR	*p*	95% CI
Age	1.066	<0.001	1.053–1.081	1.058	<0.001	1.043–1.073
*Sex*						
Female	1.000	–		1.000	–	–
Male	2.576	<0.001	1.949–3.404	1.393	0.052	0.997–1.947
*IMD*						
Most deprived	1.000	–		1.000	–	–
4	0.962	0.846	0.649–1.424	1.042	0.840	0.699–1.554
3	0.649	0.056	0.417–1.011	0.775	0.267	0.494–1.216
2	0.618	0.021	0.410–0.931	0.805	0.310	0.529–1.224
Most affluent	0.521	0.004	0.333–0.815	0.688	0.108	0.435–1.086
Unknown	–	–	–	–	–	–
Year	0.900	0.003	0.839–0.964	0.902	0.004	0.841–0.969
*Diagnosis*						
Colorectal	1.000	–	–	1.000	–	–
Anal	0.692	0.547	0.209–2.290	1.005	0.994	0.300–3.368
Bladder	1.669	0.110	0.891–3.129	1.063	0.853	0.558–2.025
Brain	0.760	0.542	0.314–1.838	1.984	0.143	0.793–4.966
Breast	0.126	<0.001	0.073–0.218	0.248	0.000	0.135–0.457
Cervical	0.380	0.112	0.115–1.251	0.810	0.737	0.237–2.769
Lung	1.388	0.204	0.837–2.302	1.283	0.345	0.765–2.152
H + N	1.441	0.126	0.902–2.304	1.837	0.014	1.133–2.978
Multiple	0.458	0.003	0.274–0.768	0.382	0.000	0.222–0.656
Oesophageal	2.166	0.032	1.071–4.383	1.893	0.091	0.904–3.967
Prostate	0.076	<0.001	0.027–0.215	0.076	0.000	0.027–0.218
Sarcoma	0.261	0.188	0.035–1.928	0.382	0.347	0.051–2.837
Uterine	0.231	0.046	0.055–0.972	0.285	0.092	0.066–1.229
Other	0.426	0.032	0.195–0.931	0.796	0.581	0.354–1.792

**Fig. 2:**
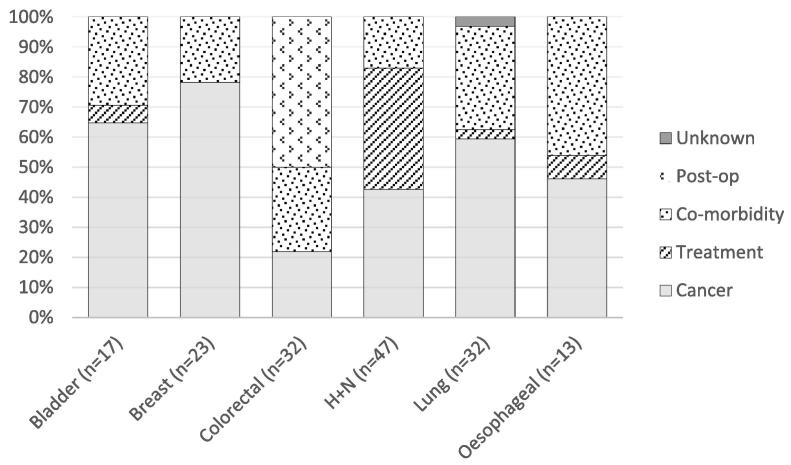
Proportion of deaths within 90 days of the start of non-palliative radiotherapy attributable to various causes.

Amongst patients who died within 90 days of the start of radiotherapy for rectal cancer 50.0% of deaths were attributed to post-operative complications, 28.1% related to comorbid disease and 21.9% disease progression. In contrast, in breast cancer, 78.3% were due to disease progression and 21.7% co-morbidity. 59.4% of lung cancer and 46.2% of oesophageal cancer deaths were due to disease progression with 34.4% and 46.2% due to co-morbidity respectively. H + N cancer was the only diagnosis in which significant numbers of deaths could be attributed to the treatment delivered (40.4%) with a further 42.6% due to the primary diagnosis and 17.0% co-morbidity (Fig. 2).

Univariable logistic regression in the H + N cancer population demonstrated that older age, non-oropharyngeal primary and earlier year of treatment were significantly associated with higher rates of 90 DM (*p* < 0.01). Age (OR 1.037 (CI 1.009–1.065)) and year of treatment (OR 0.788 (CI 0.668–0.929) maintained this significance in multivariable analysis (see Table 3). Within the H + N cancer population the average TTFT reduced from 67.8 days in 2004 to 40.1 days in 2007, this was significant on one-way ANOVA (*p* < 0.001). Further improvement in waiting times is known to have occurred in the latter part of the cohort. It is not possible to quantify this.

**Table 3:** Univariable and Multivariable logistic regression assessing the impact of variables upon 90 DM from the start of non-palliative radiotherapy for head and neck cancer.

	Univariable logistic regression			Multivariable logistic regression		
	OR	*p*	95% CI	OR	*p*	95% CI
*Subsite*						
Oropharyngeal	1.000	–	–	1.000	–	–
Other	3.689	0.014	1.307–10.410	2.674	0.070	0.922–7.750
Age	1.045	0.002	1.017–1.073	1.038	0.007	1.010–1.067
*Sex*						
Female	1.000	–	–	1.000	–	–
Male	1.294	0.483	0.630–2.656	1.424	0.348	0.680–2.982
Year	0.779	0.003	0.660–0.919	0.781	0.005	0.657–0.928
*Treatment intent*						
Adjuvant	1.000	–	–	1.000	–	–
Radical	0.834	0.584	0.435–1.598	0.785	0.487	0.396–1.554
*IMD*						
Most deprived	1.000	–	–	1.000	–	–
4	0.956	0.911	0.430–2.126	0.945	0.892	0.419–2.134
3	0.731	0.493	0.298–3.136	0.757	0.552	0.302–1.895
2	0.217	0.042	0.049–4.146	0.233	0.054	0.053–1.028
Most affluent	0.885	0.79	0.360–5.156	0.913	0.846	0.364–2.291
Unknown	Omitted			Omitted		

In lung cancer age and sex were the only factors having a significant association with 90 DM on univariable analysis. Both maintained their significance on multivariable analysis (age, OR 1.068, *p* = 0.003 (1.022–1.117), male, OR 3.327, *p* = 0.006 (95%CI 1.412–7.841) with Small-cell morphology also being associated with significantly worse 90 DM (OR 3.592, *p* = 0.004 (95%CI 1.500–8.603) (see table 4).

**Table 4:** Univariable and multivariable logistic regression assessing the association between 90 DM and the characteristics of the non-palliative lung cancer population.

	Univariable logistic regression			Multivariable logistic regression		
	OR	*p*	95% CI	OR	*p*	95% CI
*Morphology*						
NSCLC	1.000	–	–	1.000	–	–
Small cell	2.157	0.071	0.935–4.977	3.427	0.008	1.379–8.517
Unknown	2.082	0.135	0.796–5.443	1.890	0.208	0.702–5.086
Age	1.049	0.021	1.007–1.093	1.065	0.007	1.017–1.114
*Sex*						
Female	1.000	–	–	1.000	–	–
Male	2.792	0.018	1.190–6.550	2.930	0.015	1.231–6.974
Year	0.940	0.488	0.788–1.120	0.921	0.358	0.772–1.098
*IMD*						
Most deprived	1.000	–	–	1.000	–	–
4	0.601	0.346	0.208–1.734	0.598	0.347	0.205–1.746
3	0.727	0.587	0.230–2.298	0.667	0.498	0.207–2.150
2	1.368	0.504	0.546–3.425	1.124	0.807	0.438–2.887
Most affluent	0.486	0.351	0.107–2.211	0.406	0.251	0.087–1.891
Unknown	Omitted			Omitted		

## Discussion

This study aimed to assess the suitability of 90 day-mortality as a clinical indicator following radiotherapy delivered in a curative setting. We report an overall 90 DM of 1.25%. Whilst consistent with other published series [14] this overall figure concealed wide variation between cancer sites, age groups and year of treatment. Further detailed analysis was required to provide information about how this might reflect the quality of treatment delivered.

In our population 90 DM of 2.59% was seen in those receiving radiotherapy for colorectal cancer. The significant contribution of post-operative mortality in this setting (50% of 90 DM) will limit the value of this indicator in informing non-surgical practice; the impact of the whole multi-disciplinary team must be recognised and indicators used that reflect these complex care pathways.

The observed 90 DM seen following breast (0.33%) and prostate (0.20%) cancer treatments are extremely low. The greatest concern for breast cancer radiation related mortality is late cardiac toxicity, many years after treatment [20]. Improved patient selection and radiotherapy techniques may reduce this, however, these are not reflected in the 90 DM indicator.

Given the heterogeneity of the population, further detailed investigation was limited to lung and H + N cancer patients as, despite using data from a large centre, over an extended time period further investigation of the oesophageal cancer, bladder cancer and brain tumour cohorts was limited by small numbers.

### Head and neck cancer

An overall 90 DM of 3.86% for H + N cancer concealed a significant reduction over the cohort period (from a peak of 6.96% in 2005 to 0.61% in 2010). COD was split predominantly between those attributed to radiotherapy based treatment (40%) and the disease itself (43%). 90 DM might therefore provide information to support service improvements, however, anatomically these tumours can pose a significant risk of mortality. As such a 90 DM of zero is unrealistic even if treatment related deaths can be eliminated.

The change in 90 DM over time in the H + N cancer population warrants further discussion; a number of changes in the clinical service may have contributed. The service moved from a stand-alone radiotherapy centre to a large, acute teaching hospital with all the medical support services this provides, prophylactic feeding tube use increased, the proportion of HPV positive oropharyngeal patients may have risen [21] and the use of induction chemotherapy varied reflecting the changing evidence base [22]. In addition, importantly, waiting time to the start of treatment reduced markedly. It is recognised that treatment delay in H + N cancers is associated with worse outcomes [23]. The changes in the use of induction chemotherapy will, however, confound any analysis of the impact of waiting time and as such this was not investigated within the logistic regression models. The temporal variation we saw is, however, clinically significant and if mirrored in between centre variation might provide valuable information to guide service change. To attribute this to any single element of the service change would, however, ignore the complex relationships and correlations that exist between these factors. The effect observed requires, further national investigation.

### Lung cancer

In lung cancer overall 90 DM was 3.56%. This is significantly lower than that reported by other groups [24], COD was predominantly disease progression (59%, with 34% attributed to comorbidity) and no significant change in 90 DM was seen over time. The observation that 90 DM in lung cancer was predominantly due to disease progression is important. Poor stage-specific lung cancer survival in England has previously been demonstrated, with suboptimal treatment usage suggested as a possible contributor [25]. Attempts to minimise 90 DM may reduce access to potentially curative treatments for those deemed to be at higher risk of early mortality, whether due to frailty, co-morbidity or disease stage. As such aiming to reduce 90 DM could undermine attempts to improve lung cancer outcomes through increased treatment access [26], [27]. Conversely, recent data support the value of intensive supportive follow-up for improving survival in lung cancer patients [28]. Further investigation is required, using national data to assess 90 DM in the context of treatment access, allowing development of complementary measures of process quality; for example, average waiting times and curative treatment rates. Such an approach may provide balance and prevent inappropriate responses to a single indicator.

These two conflicting examples highlight the varying meaning of 90 DM in different populations. The quality of supportive services might contribute in the head and neck population, whilst 90 DM in the lung cancer population may, potentially, be more representative of case selection in clinical decision making. These disease specific factors must be considered in implementing any 90 DM metric. Additionally, variation in COD, even within diagnostic groups, means this indicator will never be able to fully separate case selection from treatment effect. As such outlying results require in depth investigation to understand what is driving the effect observed. Even after adjustment for case-mix variation unmeasured confounders and failings of data capture may contribute significantly to apparently outlying results.

A final element of considering the role of 90 DM in this setting is considering its power to detect outlying results. Using H + N as an exemplar, power calculations for a one-sample, 1-sided, proportion test, with α = 0.05 (5% probability of a false positive) were calculated using Stata IC 14 [11], [29]. Assuming a national average 90 DM of 3% cohorts of at least 257 patients are needed to deliver 80% probability of identifying a Centre with twice the national average 90 DM. This number of H + N treatments annually will only be seen in the largest centres (supplementary Fig. 1s). Varying the power and type 1 error rate might allow assessment in smaller cohorts (Table 2s) but at the cost of reduced ability to detect outlying outcomes (with potential for complacency in services wrongly identified as performing within acceptable limits) or more false positives (with accompanying reputational damage, patient anxiety and investigative resource). H + N cancer is the 4th most frequently treated diagnosis in this cohort; for smaller diagnostic groups this number of treatments annually is entirely unrealistic. Robust annual comparisons of Centre performance using 90 DM is, therefore, unlikely to be possible. Aggregating diagnostic groups or time periods might provide adequate numbers, however, heterogeneity between diagnoses and changing treatment patterns over time could mean loss of clinical relevance and significant variation in one group concealed by others. This approach is unlikely to be justified, although service centralisation might contribute to clinically relevant aggregation.

We acknowledge that our investigation has a number of limitations; we report a single centre study. This large dataset allows in-depth analysis within robust local data, however, it is unclear if these rates of 90 DM will be replicated elsewhere. Additionally, currently available routinely collected data may not support this analysis due to a lack of clarity about treatment intention; work to rectify this, using linked routine datasets, is urgently needed; COD assessment is extremely challenging, the best assessment possible was made but we acknowledge this limitation; assessment of the impact of other elements of the treatment pathway, particularly concurrent chemotherapy was beyond the scope of this study but should be included in future investigations.

This study does not support the adoption of the 90 DM indicator as a centre wide measure of quality following RDCS. Further National and International research is required to identify diagnostic groups for whom this measure may have value and consider carefully the statistical validity of National comparisons in order to avoid encouraging complacency. Alternative longitudinal monitoring techniques should be considered (for example cumulative sum charts (CUSUM) [30]. These can provide case-mix adjusted, within centre monitoring, enabling early identification of deviation from an acceptable baseline or persistent outliers, triggering further investigation.

Until further work is completed to investigate this indicator, Centres should continue to monitor their own services critically, reviewing deaths within 90 days of RDCS to derive lessons for future practice.

## Conclusion

Despite the current drive to report comparative outcomes, the results of this study do not support the adoption of 90 DM as a centre wide indicator of quality in curative radiotherapy. If these findings are also found in more extensive, national/international studies the 90 DM indicator may have value only in specific groups. Identifying which, if any, these are and how statistically valid outcomes can be reported is now vital to ensure that the, much needed, clinical indicators developed provide meaningful comparisons, able to facilitate improvements in care.

## Conflicts of interest statement

KS, RE, ED, RB, RT, GH, EM and DSM have no conflicts of interest to declare. AC is the National Clinical Lead for Proton therapy for NHS England.

## Funding source

Whilst undertaking this work KS was funded through the Wellcome Trust Institutional Strategic Support Fund at the University of Leeds and subsequently a Medical Research Council Clinical Research Training Fellowship (MR/N021339/1). EM was funded by the Leeds Medical Research Council Medical Bioinformatics Centre (MR/L01629X/1). RB was funded by a Cancer Research UK Population Health Fellowship (C34080/A16438). The study sponsors had no involvement in the study design, in the collection, analysis and interpretation of data; in the writing of the manuscript; or in the decision to submit the manuscript for publication.
