# Supplementary material for: Caution is required in the implementation of 90-day mortality indicators for radiotherapy in a curative setting: A retrospective population-based analysis of over 16,000 episodes
Source: Radiother Oncol. 2017 Oct;125(1):140–6. doi: 10.1016/j.radonc.2017.07.031 (PMC5648077; doi:10.1016/j.radonc.2017.07.031)
Supplement: Supplementary data [file mmc1.pdf]

Figure 1s. Expected number of radical treatment courses for head and neck cancer delivered by English radiotherapy centres based on number of radical treatments within each centre and same proportion of head and neck cancer treatments as seen in the study cohort.

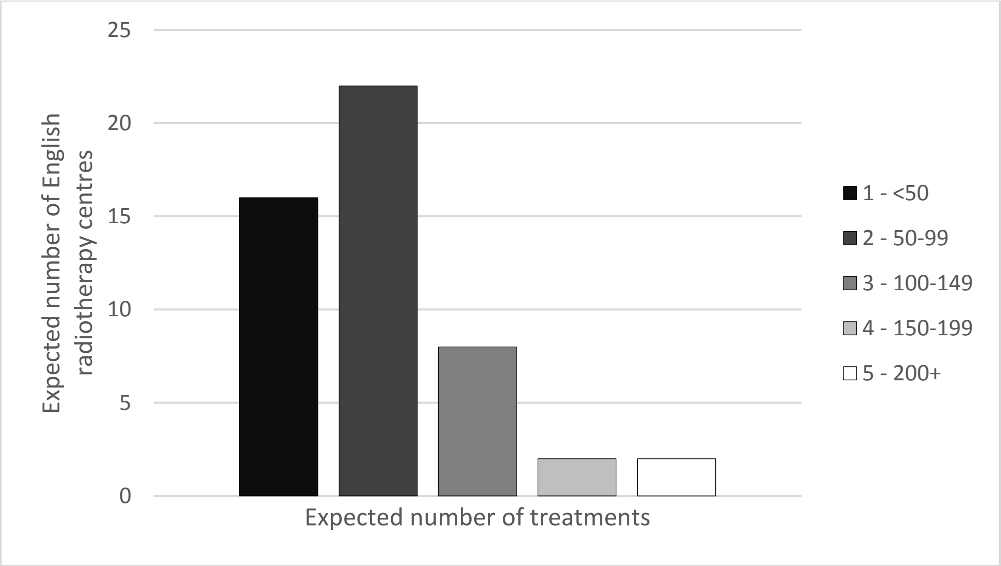

Table 1s. Allocation of ICD-10 coded diagnoses to diagnostic groups for analysis.

**Diagnostic group**	**ICD-10 codes**
**H+N**	C00-C14
	C30-C32
**Oesophageal**	C15
**Colorectal**	C17-C20
**Anal**	C21
**Lung**	C33-C34
**Breast**	C50
**Prostate**	C61
**Bladder**	C65-C67
**Brain**	C70-C71
**Cervical**	C53
**Sarcoma**	C40-41
	C46-C47
	C49
	C72
**Uterine**	C54-C55
**Other**	C16
	C22-C24
	C25
	C26
	C37-C39
	C43
	C45
	C48
	C51-C52
	C56
	C57
	C60
	C62-C64
	C68-C69
	C73
	C74-C77
	C78-C80
**Skin**	C44
**Haematological**	C42
	C81-C85
	C88
	C90-C92
	C95-C96

Table 2s. Sample sizes required for varying levels of power to identify outlying outcomes given differing levels of acceptance of false positives (type I errors).

**Type I error rate α**	**Power (1-β)**			
	**60%**	**70%**	**80%**	**90%**
**0.025**	173	234	318	454
**0.05**	130	183	257	381
**0.1**	87	131	195	304
